# Supplementary figures and images for: Insular holobionts: persistence and seasonal plasticity of the Balearic wall lizard (Podarcis lilfordi) gut microbiota
Source: PeerJ. 2023 Jan 3;11:e14511. doi: 10.7717/peerj.14511 (PMC9817956; doi:10.7717/peerj.14511)

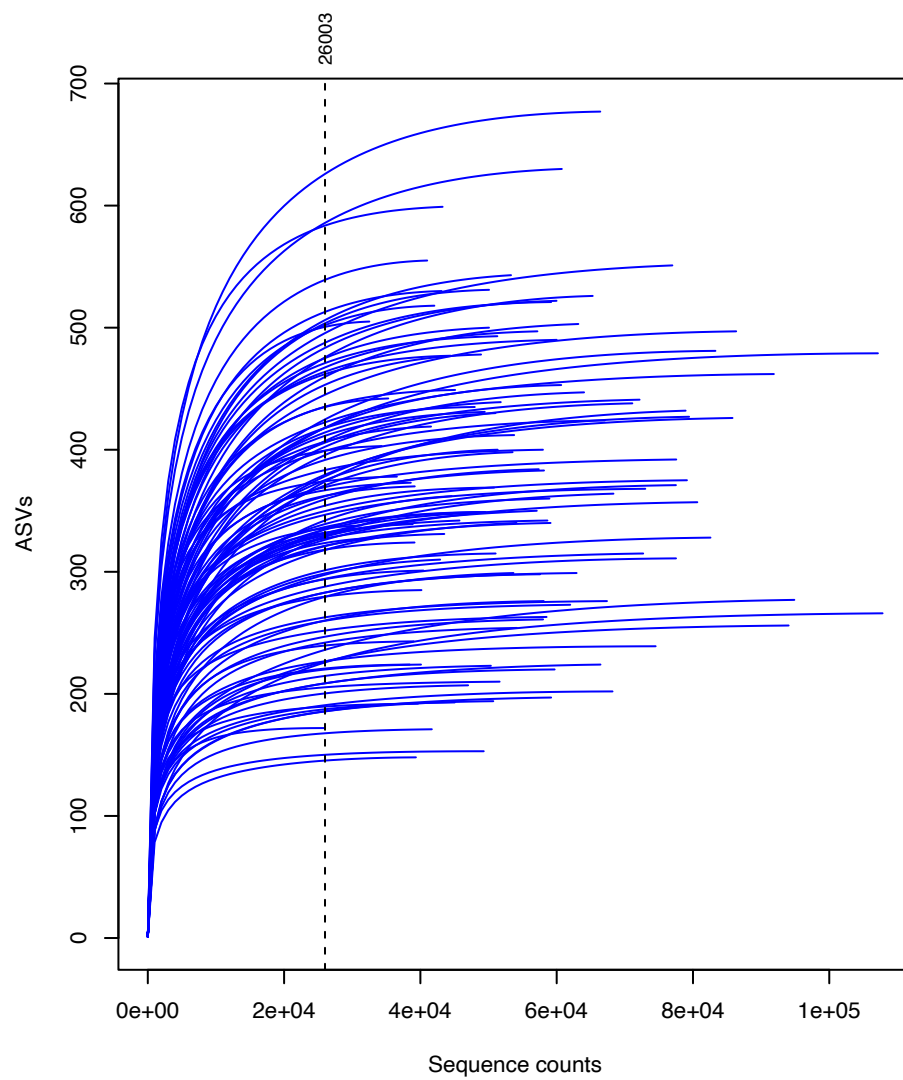

Supplement: Supplemental Information 8 — The dashed line shows the sample with minimum sequence coverage (260003 sequence counts). [file peerj-11-14511-s008.pdf]

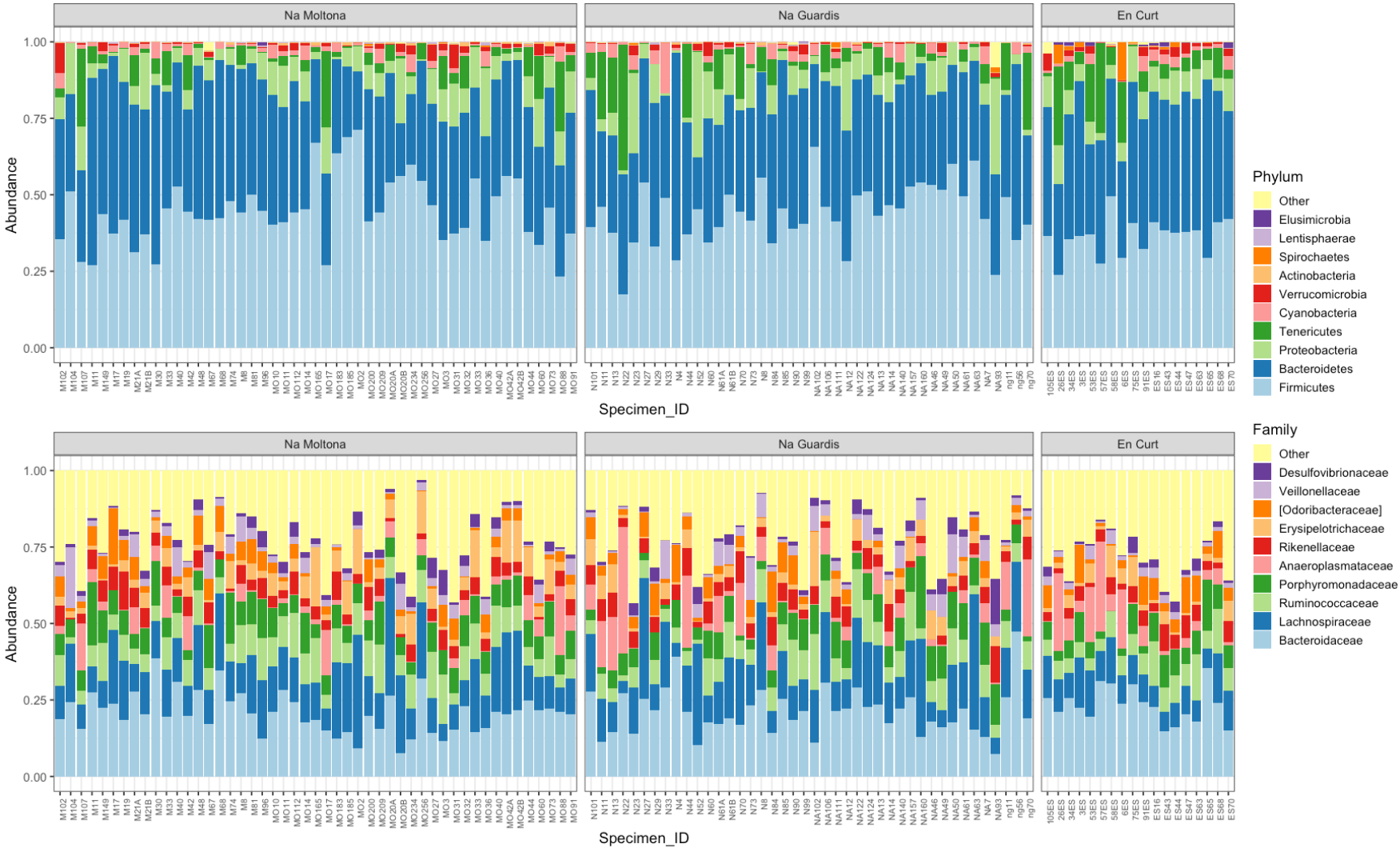

Supplement: Supplemental Information 9 — Legends list only the top ten most abundant taxa. The remaining were included in “Others”. [file peerj-11-14511-s009.pdf]

Na Guardis

Na Moltona

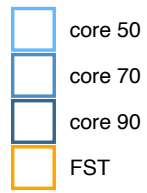

En Curt

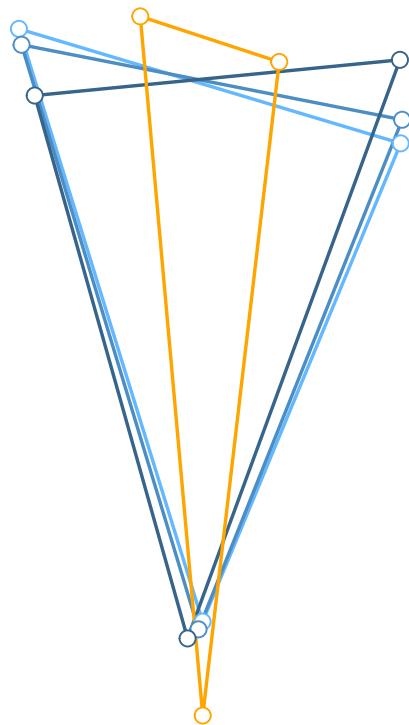

Supplement: Supplemental Information 10 [file peerj-11-14511-s010.pdf]

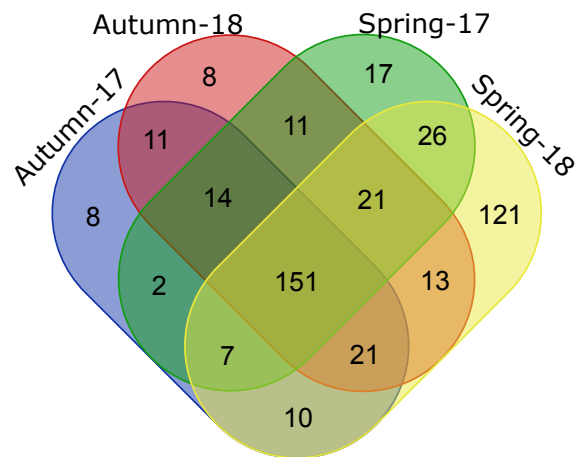

NM

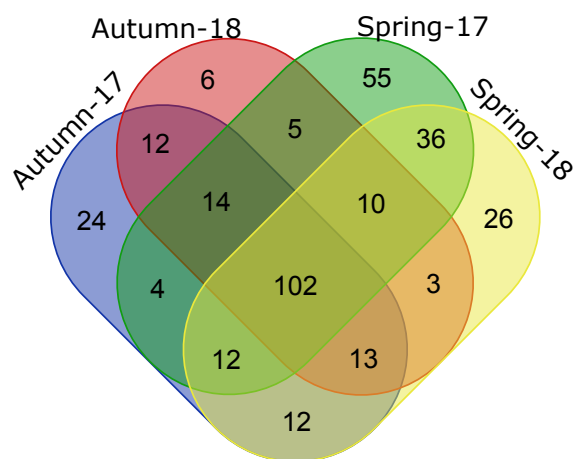

NG

Supplement: Supplemental Information 11 — For each date, we considered only ASVs found in at least 50% of the specimens. [file peerj-11-14511-s011.pdf]

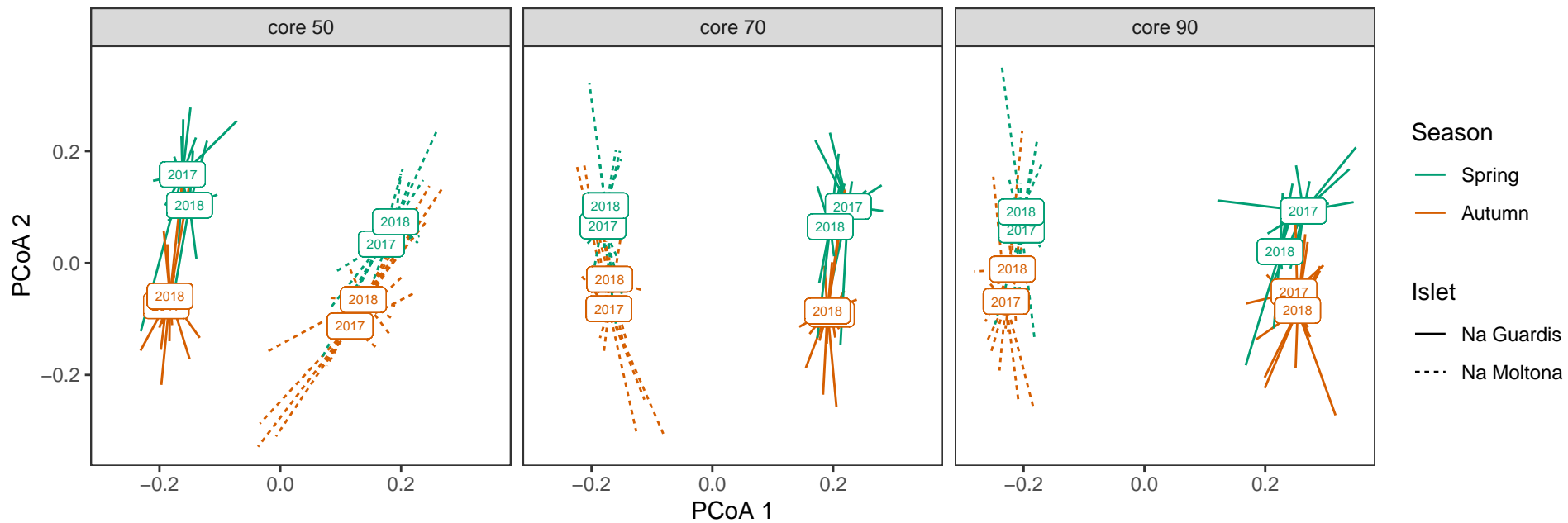

Supplement: Supplemental Information 12 — The rectangular box represents the centroid per date. [file peerj-11-14511-s012.pdf]

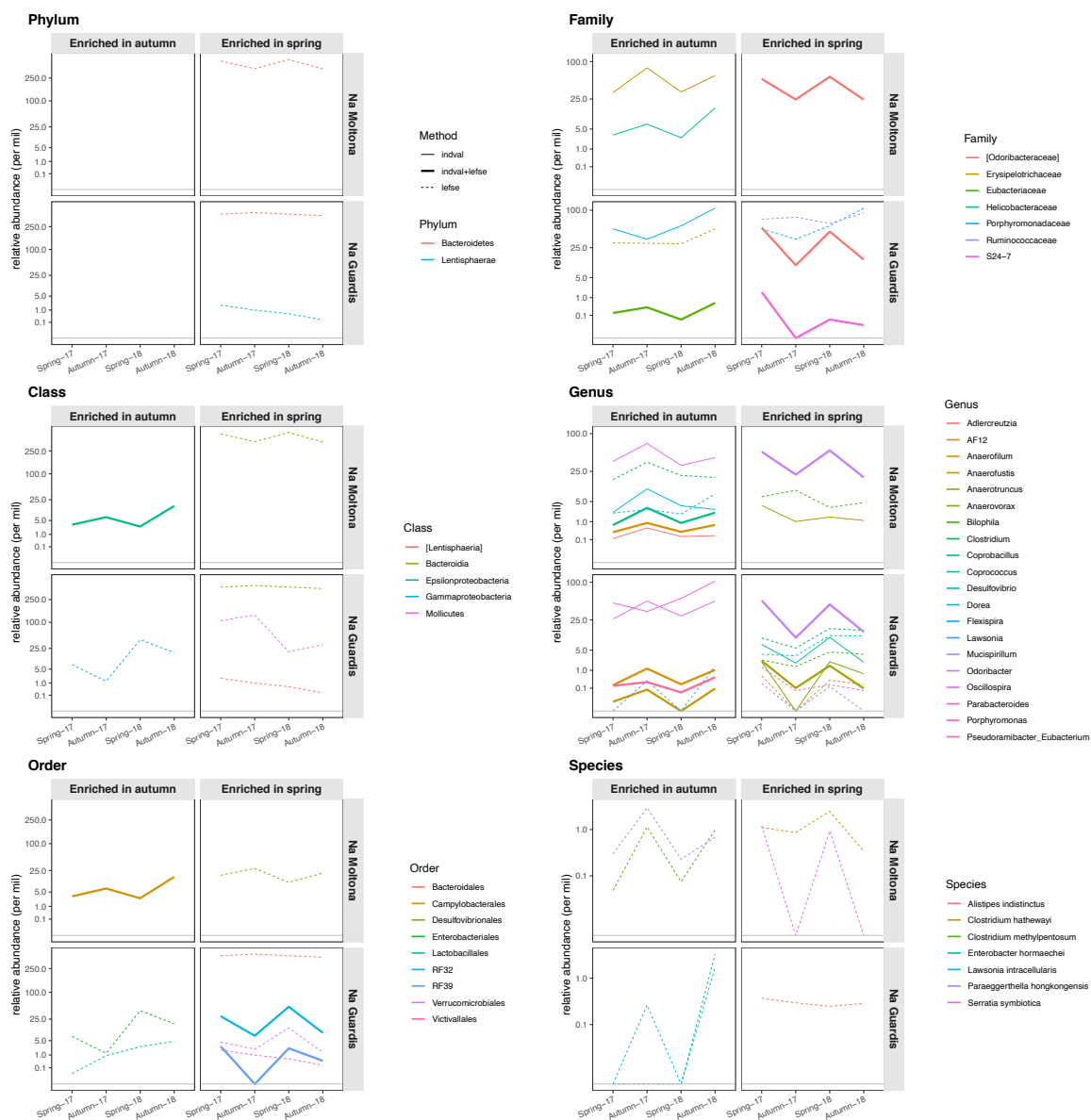

Supplement: Supplemental Information 13 — Fluctuations in relative abundance between dates for all taxonomic levels (Species to Phylum) were calculated as mean relative abundances on reads aggregated by levels (i.e., after adding all all reads corresponding to a particular taxon). [file peerj-11-14511-s013.pdf]
